# Evolving trends in psychiatric emergency services in Southern China: a seven-year retrospective analysis

**DOI:** 10.1186/s12245-025-00904-5

**Published:** 2025-06-18

**Authors:** Cuiling Zhang, Suiyun Weng, Xiaoyu Zhang, Songkang Liu, Min Yu, Miaoling Jiang

**Affiliations:** 1 Department of Outpatient and Emergency Department, National Clinical Key Specialty, The Affiliated Brain Hospital, Guangzhou Medical University, Guangzhou, 510370 China; 2 Key Laboratory of Neurogenetics and Channelopathies of Guangdong Province and the Ministry of Education of China, Guangzhou Medical University, Guangzhou, China; 3 College of Nursing, Guangzhou Medical University, Guangzhou, China

**Keywords:** Psychiatric emergency services, Evolving trends, Mental health services, Retrospective analysis, China

## Abstract

**Background:**

The Affiliated Brain Hospital, Guangzhou Medical University is an important provider of psychiatric emergency services (PES) in southern China. Revealing the evolution trend of the psychiatric emergency services of this hospital can help decision-makers formulate relevant policies. However, at present, there is a lack of large-scale, long-term retrospective studies.

**Methods:**

A retrospective analysis was conducted on patient records from the psychiatric emergency room (PER) of the Affiliated Brain Hospital, Guangzhou Medical University. Data included demographic and clinical variables were aggregated annually and described using percentages from 2018 to 2024. Chi-square and Fisher’s exact test were used to confirm significance of the trends.

**Results:**

More voluntary health-seeking behaviors, broader medical insurance coverage, more cautious use of restraint measures, and more precise diagnoses were observed from 2018 to 2024. Besides, there were an increasing number of younger, highly educated, unmarried, and unemployed visitors. We also found that the gender gap is widening and medical resources are increasingly strained. There are differences between judicial and medical personnel in making compulsory decisions. During Covid-19, the demographic and clinical variables show significant changes.

**Conclusions:**

PES in southern China have developed to a certain extent, but they are also confronted with obstacles at the same time. These trends underscore the need for enhanced referral systems, expanded community-based psychiatric care, ethical guidelines for managing coercive measures and strengthening the response strategies for public event crises.

Mental disorders remain a leading contributor to the global disease burden [1]. The COVID-19 pandemic has further exacerbated this crisis, with anxiety and depressive disorders rising by over 25% during its first year [2]. This unprecedented global health emergency has disrupted mental health services worldwide, intensifying disparities in accessing timely treatment [2]. For individuals with acute and severe psychiatric conditions, psychiatric emergency services (PES) play a crucial role in providing immediate care and ensuring safeguarding public safety. Consequently, numerous hospitals have established dedicated psychiatric emergency rooms (PERs) to provide round-the-clock emergency care.

As a populous country, China faces unique challenges and opportunities in the field of mental health services. Mental health services in China have developed relatively late compared to other nations. The first psychiatric hospital in Guangzhou, established by John Kay in 1898 [3], marked the beginning of specialized mental health care in the region. Currently, this hospital has been renamed as the Affiliated Brain Hospital, Guangzhou Medical University, one of the largest and oldest psychiatric hospitals in China.

PES have made remarkable progress since China enacted the Mental Health Law of the People’s Republic of China in 2013 [4], which encouraged the establishment of PER across the country. The Affiliated Brain Hospital, Guangzhou Medical University has became one of the first mental health specialty hospitals to establish the PER. The hospital is located in Guangzhou, the capital city of Guangdong Province. It not only takes charge of receiving mental health patients from within Guangdong Province but also undertakes the diagnosis and treatment of the patients referred from the Southern China. Due to its unique geographical location, rapid economic development and diverse cultural background, it is typical and representative in the construction of PES system.

The characteristics and utilization of PES are influenced by local public health systems and broader societal factors [5, 6], making them a key area of investigation in mental healthcare research. One study [6] in Boston examined the impact of the Boston Marathon bombings on PES, highlighting how external crises can drastically affect patient encounters. In the Netherlands, a comparative analysis of psychiatric emergency consultations between 1983 and 2004–2005 revealed an expansion of services for acute psychiatric situations, reflecting evolving public mental health needs [7]. Research in Saskatoon, Saskatchewan, explored patterns in PES utilization, underscoring regional differences [8]. Additionally, studies have focused on specific populations, such as children [9], asylum seekers [10], and individuals with suicidal behaviors [11], demonstrating the diverse demands placed on PES. External factors, including severe weather, public holidays, unemployment, and income schedules, have also been shown to influence PES workloads, highlighting the dynamic nature of psychiatric emergencies [12].

In our previous research [13], we conducted an analysis of the seasons and specific time points during which psychiatric patients sought emergency treatment across a span of three years. Our findings revealed a strong temporal correlation in the behavior of emergency patients seeking care within the psychiatry department.

Studying the evolution trend of the PES at the Affiliated Brain Hospital, Guangzhou Medical University not only provides valuable practical experience for local health policy makers and mental health professionals, but also offers references for the development of mental health services in other regions and even globally. However, there is a lack of large-scale, long-term retrospective studies on the evolution trends of mental emergency services in southern China.

To gain a more comprehensive and detailed understanding, we integrated data from 7 years before and after the pandemic. This broader dateset allowed us to further uncover the temporal variation patterns in emergency care-seeking behavior within psychiatry and to elucidate the evolving trends in PES. The findings will contribute to the development of evidence-based strategies to enhance service quality, improve resource allocation, and promote early intervention.

## Methods

### Study design and setting

This study employed a retrospective analysis of patient records collected from the PER of the Brain Hospital of Guangzhou Medical University in Guangdong, China.

### Participants

#### Inclusion criteria

Patients who visited to the PER in the Affiliated Brain Hospital, Guangzhou Medical University between January 1, 2018, and December 31, 2024.

#### Exclusion criteria

Registered patients who did not complete a consultation with a physician. Patients with more than 30% missing data in their medical records related to PER visits.

#### Data collection

Data for this study were extracted from the iMedical Total HIS (Health Information System) version 8.2.0, utilized by the Affiliated Brain Hospital, Guangzhou Medical University. The datasets includes records of PER visits from 2018 to 2024. Variables collected were categorized into the following:

- *Demographic variables*: gender, age, marital status, occupation, education status and insurance coverage.
- *Clinical variables*: emergency triage level, use of restraint measures, health-seeking behavior, types of admission, emergency outcomes, preliminary first diagnoses and the escorts to the hospital. Diagnoses were classified according to the International Classification of Diseases, 10th Revision (ICD-10) [14].

The classification of psychiatric emergency triage level is based on the the rating scales of violence and suicide, which were recommended by the Provincial Ministry of Health (MOH) [15]. Psychiatric emergency triage was categorized patients into four levels:

- *Life-threatening*: Conditions such as shock, cardiac arrest, coma, life-threatening organic diseases, or a current risk score of 5 for violence or suicide.
- *Urgent conditions*: Acute drug poisoning, epilepticus, or a current risk score of 4 for violence or suicide.
- *Semi-urgent conditions*: Excitement, agitation, behavioral disorders, or a current risk score of 2–3 for violence or suicide.
- *Low-urgency conditions*: Hallucinations, delusions, sleep disorders, anxiety, or a current risk score of 0–1 for violence or suicide.

### Data analysis

Data analysis was performed using WPS EXCEL 2024, which aggregated demographic variables and clinical information annually and described using percentages. The chi-square (χ^2^) test was employed to analyze the distribution differences of demographic and clinical data across the years 2018–2024, with statistical significance defined as a two-tailed *P* < 0.05. All statistical analyses were performed using SPSS 21.0 software.

## Results

### Evolving trends in demographic characteristics of PER visits

**Fig. 1:**
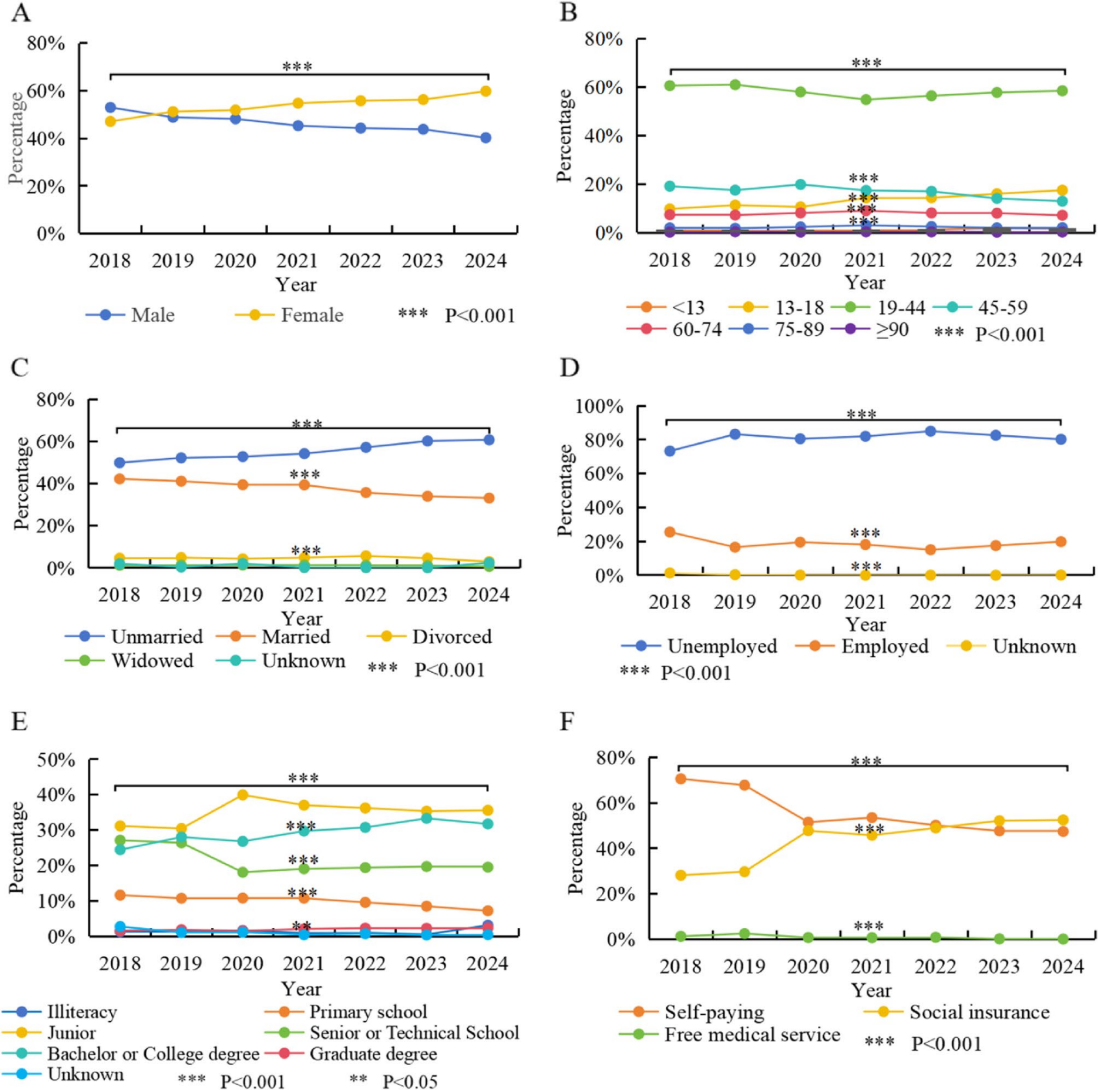
Evolving Trends and statistical differences in Demographic Characteristics of PER Visits from 2018–2024. **A** Gender Distribution. **B** Age Distribution. **C** Marital Status Distribution. **D** Occupation Status Distribution. **E** Education Status Distribution. **F** Insurance coverage Distribution. Notes: Line graph showing discrete yearly data points (solid circles) for each variable, connected by straight lines to illustrate temporal trends. Different colors line represent different variables. Each point represents the percentage value of different variables in a specific year. Significant differences between time points identified by chi-square tests are marked with asterisks: The proportion of female, adolescent patients (under 18 years old), unmarried, unemployed, highly educated (Bachelor or College degree), and those with medical insurance coverage seeking medical treatment has also risen (*P* < 0.001). The proportion of patients aged 45–49 seeking medical treatment has decreased (*P* < 0.001). During the period of 2020–2021, there were significant changes in the gender distribution, age distribution, educational attainment distribution and the proportion of medical insurance visits among emergency patients compared with 2019 (*P* < 0.001)

Our research encompassed a total of 42,846 emergency patients, among whom 19,242 (44.91%) were male and 23,604 (55.09%) were female. From 2018 to 2024, the number of psychiatric emergency visits was 3,111, 3,501, 5,170, 7,808, 6,045, 7,397, and 9,814 respectively. Overall, the trend showed an increase from 2018 to 2024, with a notable rise in 2021, a decline in 2022, and another increase from 2023 to 2024. As depicted in Fig. 1A, with respect to the gender distribution of the visited patients, the gender disparity has been widening. The proportion of male visitors decreased from 52.94% in 2018 to 40.20% in 2024 (χ^2^ = 216.422, *P* < 0.001).

Among all the emergency visits to the psychiatry department, the top three age groups in terms of proportion were the 19–44 age group (57.60%), the 45–59 age group (16.20%), and the 13–18 age group (14.33%). Nevertheless, over the past seven years, the age of the patients seeking treatment has gradually become younger. As depicted in Fig. 1B, the proportion of the 13–18 age group rose from 9.77% in 2018 to 17.50% in 2024 (χ^2^ = 50.227, *P* < 0.001). Meanwhile, the proportion of the 45–59 age group presented a downward trend, decreasing from 17.2% in 2018 to 12.96% in 2024 (χ^2^ = 186.653, *P* < 0.001).

Among all the emergency department visits for mental health issues, a higher proportion of unmarried individuals (56.50%) and unemployed patients (81.35%) were observed. In terms of the temporal trend, from 2018 to 2024, the overall proportions of unmarried (48.89% vs. 60.74%) and unemployed patients (73.22% vs. 80.17%) both showed an increasing trend (χ^2^ = 241.725, *P* < 0.001 vs. χ^2^ = 190.382, *P* < 0.001), as illustrated in Fig. 1C and D.

In terms of educational attainment, the top three educational levels of the respondents were junior education (35.66%), bachelor’s or college degree education (29.99%), and senior or technical education (20.39%). As shown in Fig. 1E, the educational level of the patients gradually increased, with the proportion of those holding a Bachelor or College degree rising from 24.43% in 2018 to 31.72% in 2024 (χ^2^ = 131.379, *P* < 0.001). However, there was a special growth trend in 2020, where the number of visitors to junior high education (39.90%) significantly increased.

As shown in Fig. 1F, in terms of the proportion of medical expenses paid, although the overall proportion of self-paying patients is relatively high (52.83%), the coverage rate of medical insurance has gradually increased in recent years. The proportion of medical insurance payments has risen from 28.13% in 2018 to 52.52% in 2024 (χ^2^ = 1077.157, *P* < 0.001).

### Evolving trends in clinical characteristics of PER visits

**Fig. 2:**
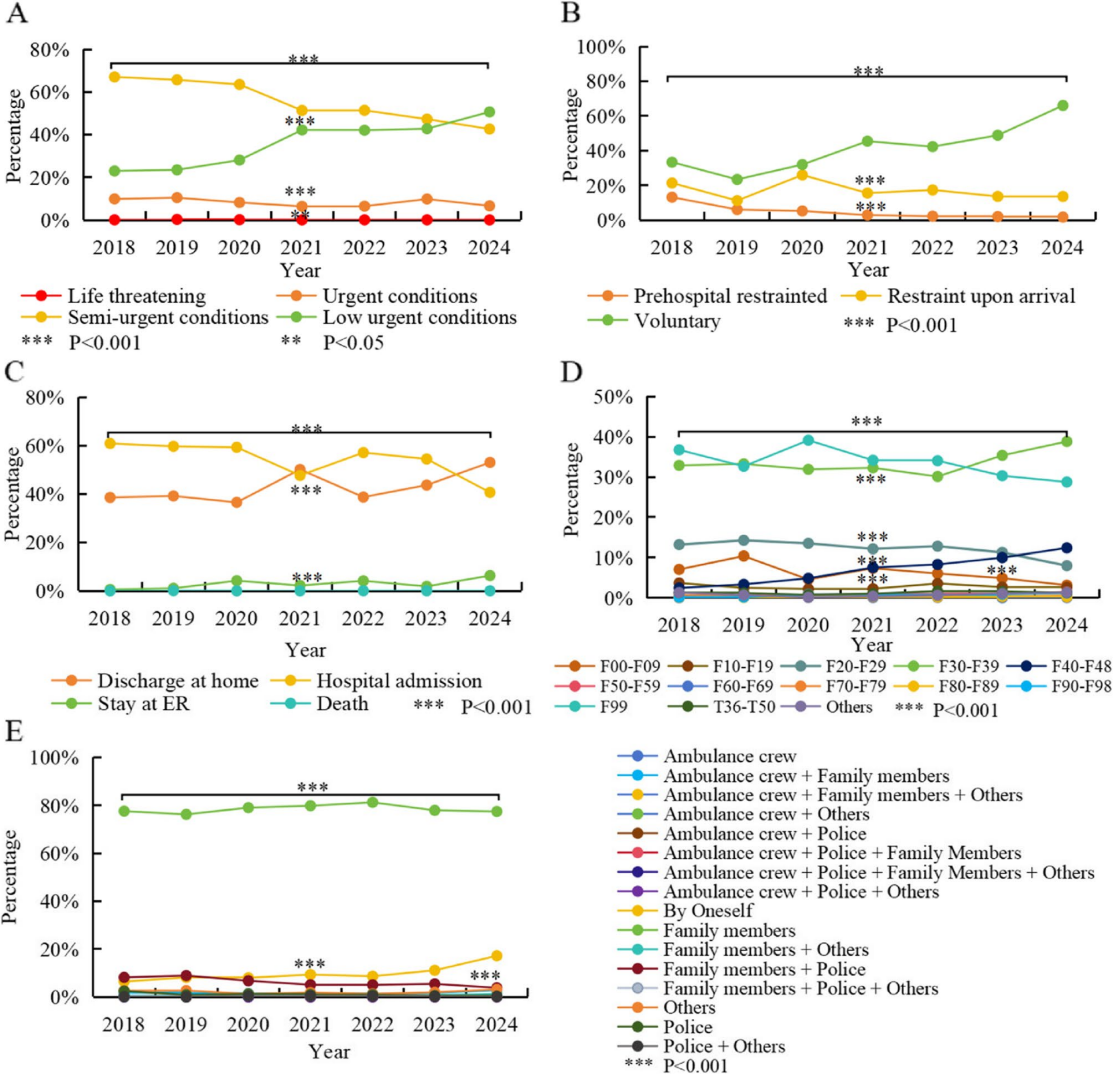
Evolving Trends and statistical differences in Clinical Characteristics of PER Visits from 2018–2024. **A** Emergency Triage Distribution. **B** Health-seeking Behavior and Restrictive Measures Used Distribution. **C** Emergency Outcomes Distribution. **D** Diagnostic Categories Distribution. **E** Escort Distribution. Notes: Line graph showing discrete yearly data points (solid circles) for each variable, connected by straight lines to illustrate temporal trends. Each point represents the percentage value of different variables in a specific year. Significant differences between time points identified by chi-square tests are marked with asterisks. The proportion of voluntary health-seeking behaviors and the low-level triage trend has increased, while proportion of the restraint measures used and the admission to the hospital have shown opposite trends (*P* < 0.001). The diagnoses has become more precise with less patients diagnosed as F99 (*P* < 0.001). Most patients were escorted by family, but came by oneself were increased (*P* < 0.001). During the period of 2020–2021, compared with 2019, there were significant differences in the emergency triage distribution, the usage of protective restraint, the proportion of patients seeking medical treatment voluntarily and inpatients patients, as well as the diagnoses distribution (*P* < 0.001)

Among all the included psychiatric patients, the triage level of Semi-urgent conditions accounted for the highest proportion (52.46%), followed by Low urgent conditions (39.60%). As depicted in Fig. 2A, however, the proportion of the low-level triage trend gradually increased, from 22.98% in 2018 to 50.68% in 2024 (χ^2^ = 1596.906, *P* < 0.001).

In terms of health-seeking behavior, although the proportion of involuntary patients remains relatively high at 53.97%, as shown in Fig. 2B, the proportion of voluntary patients has risen from 33.40% in 2018 to 66.03% in 2024 (χ^2^ = 2965.729, *P* < 0.001). Additionally, in recent years, a more cautious approach has been adopted in the use of restrictive measures. The rate of pre-hospital restraint has dropped from 13.23% in 2018 to 1.77% in 2024 (χ^2^ = 539.392, *P* < 0.001). The rate of restraint after admission has also decreased from 21.41% in 2018 to 13.65% in 2024 (χ^2^ = 538.346, *P* < 0.001). However, the post-admission restraint rate has consistently been higher than the pre-hospital restraint rate.

Regarding emergency outcomes, among the included psychiatric patients, 51.91% of the visitors were admitted for inpatient treatment, while 44.68% of them received home-based care. Overall, as illustrated in Fig. 2C, the proportion of hospital admissions has exhibited a declining trend (χ^2^ = 940.341, *P* < 0.001), while for the that who received home-based care has shown an opposite trend (χ^2^ = 692.432, *P* < 0.001). In 2021, the proportion of hospital admissions and emergency observation in 2021 decreased significantly.

Of all the visitors included, the most prevalent diagnostic categories among visitors were mood disorders (F30–F39) at 34.08%, unspecified mental disorders (F99) at 32.90%, schizophrenia and related disorders (F20–F29) at 11.54%, neurotic, stress-related, and somatosensory disorders (F40–F48) at 8.14%, and organic mental disorders (F00–F09) at 5.68%. As shown in Fig. 2D, the trend in diagnoses is gradually becoming more precise, with a notable shift in proportions over time. Specifically, the proportion of F99 diagnoses has decreased from 36.74% in 2018 to 28.74% in 2024 (χ^2^ = 220.434, *P* < 0.001). Meanwhile, diagnoses within the F40-F48 category have increased from 2.48 to 12.37% (χ^2^ = 585.914, *P* < 0.001). Diagnoses for F20-F29 have shown a gradual decline (χ^2^ = 185.237, *P* < 0.001)., while those for F30-F39 have risen from 32.88% in 2018 to 38.76% in 2024 (χ^2^ = 171.68, *P* < 0.001).

Among individuals accompanying patients to medical appointments, the predominant group consists of family members (85.90%). Additionally, 10.58% of patients attend their appointments independently, 7.09% are escorted by law enforcement personnel, 2.22% are transported by ambulance staff, and 3.30% fall into the other category. As illustrated in Fig. 2E, in recent years, the proportion of patients arriving at the hospital independently has gradually increased from 6.36 to 16.06% (χ^2^ = 7.685, *P* = 0.247), while the proportion of those accompanied by family members (χ^2^ = 226.16, *P* < 0.001) or family members and police (χ^2^ = 212.146, *P* < 0.001) has shown a gradual decline.

## Discussion

### Positive trajectory of PES in Southern china: unraveling the growth facets

PES in southern China have demonstrated substantial progress between 2018 and 2024, as evidenced by more voluntary health-seeking behaviors, broader coverage of medical insurance, more cautious restraint measures used, and more precise diagnoses.

The observed increase in autonomous medical-seeking behavior reflects two synergistic drivers. One is improving self-medical-seeking behavior through mental health education [16]. The other is the rapid proliferation of mental health apps that facilitate self-assessment and care navigation [17]. The Chinese government has made significant investments in healthcare infrastructure and expanded health insurance programs throughout the past ten years [18]. Specifically, insurance enrollment rates increased from 28.13% (2018) to 52.51% (2024) in our study cohort, significantly improving PES accessibility for economically vulnerable populations [19].

The restraint rate and the proportion of individuals accompanied by family members and police have gradually diminished. This phenomenon can be attributed to the recent establishment of clear guidelines and comprehensive policies by China concerning the standardized implementation of compulsory medical treatment [20].

The proportion of emergency psychiatric patients diagnosed with F99 has decreased from 36.74 to 28.74% (2018–2024). This trend indicates enhanced diagnostic accuracy and the broader adoption of advanced tools. Concurrently, the National Health Commission and the Ministry of Education have expanded the number of universities offering undergraduate psychiatry programs to address the increasing demand for qualified professionals in this field [21]. Furthermore, since 2020, the Affiliated Brain Hospital, Guangzhou Medical University has implemented a comprehensive training program for chief residents, mandating that all psychiatric emergency physicians rotate through inpatient psychiatric wards and pass rigorous assessments. These initiatives have fostered more precise and standardized diagnostic practices while improving overall quality in psychiatric care.

Of all the visitors included, the most prevalent diagnostic categories among visitors were F30–F39, F99 and F20–F29. In contrast, conditions like mental and behavioral disorders due to F10–F19, including alcohol and cannabis abuse, or F60–F69 are more frequently observed in other nations [5, 22–24]. This difference is likely attributed to China’s stricter regulation of psychoactive substances, which may limit the prevalence of related psychiatric emergencies.

### Impediments and ethical quandaries hampering PES in Southern China

PES in southern China are facing the following predicaments: an increasing number of younger, highly educated, unmarried, and unemployed visitors; a widening gender disparity; increasingly strained medical resources; and heightened vulnerability to the impact of public health events. Besides, there are differences between judicial and medical personnel in making compulsory decisions, which may involve ethical issues.

We observed that patients seeking treatment have become younger, and more educated. This may be because teenagers are still developing, making their cognition more vulnerable to external influences. Their psychological resilience tends to be relatively weak, and they often encounter significant academic pressure and challenges in peer relationships [25]. Two-stage intervention measures may be needed to identify those in need and then engage them in the health-care system [26]. Schools should introduce educational psychologists to conduct evaluations, prevention, and interventions, creating an environment that promotes emotional health and prevents psychological problems [27]. It is also necessary to establish mental health screening centers staffed by professionals to identify students with psychological problems at an early stage and refer them for timely treatment. Meanwhile, social media can play a significant role in promoting mental health in adolescents [28]. It is crucial to guide teenagers and adolescences in using social media responsibly. Enhancing social support can also prevent the occurrence of mental health disorders among college students [29]. Effective guidance on social media use helps teenagers manage platforms responsibly. Strengthening social support also prevents mental health issues among college students.

The overall proportions of unmarried and unemployed patients both showed an increasing trend. In addition to the changes in the age structure of patients, we believe that employment conditions are closely related to mental health, similar to the findings of a study conducted in Spain [30]. The Chinese government should promote employment stability through policy. Social platforms like TikTok or WeChat could facilitate interactions and community engagement, reducing social isolation among unmarried individuals.

Female patients account for more visits, and the gender gap is widening. A study in Chile also found that phenomenon [31]. A prior study found that women are more likely to overcome their negative attitudes towards psychological services and seek help actively [32]. Due to prevailing masculine norms and the public’s insufficient awareness of the symptoms of male mental health disorders., men tend to underutilize mental health services [33, 34]. We propose that future researchers in psychiatric emergency care should pay attention to the obstacles patients encounter when seeking medical treatment. Meanwhile, among all emergency visitors, mood disorders (F30-F39) were the most common diagnosis, partly due to the prevalence and gender differences in mental disorders presentation. Studies have shown that the likelihood of women experiencing major depressive episodes (MDE) is about 1.7 times that of men [35]. Biological factors, such as ovarian hormone fluctuations [36], increase vulnerability, while psycho-social factors, such as sensitivity to external stressors, exacerbate the risk [37]. Gender differences should be considered when diagnosing, treating, and caring for psychiatric emergency patients [38].

Our study found that post-admission restraint rates remained higher than pre-hospital rates annually, indicated that more coercive medical measures used by PER clinicians. The acute and high-pressure nature of care in the ED often gives rise to ethical conflicts, particularly regarding patient rights and more broadly in clinical decision-making [39]. And the emergency department is a clinical setting where rapid decision-making is essential to avoid potential delays or omissions in care [40]. Crowding in emergency psychiatric waiting rooms may increase the need for seclusion, restraint, or medications for agitation [41]. Mental health legislation stipulates where a patient with mental disorder has self-harming behavior or poses a danger to the safety of others or the medical order and there are no alternative measures, medical institutions and their medical staff may take protective medical measures such as restraint and isolation [4]. Emergency doctors are confronted with a highly tense situation between preventing direct harm and respecting patients’ self-determination [40]. From a principlist medical ethics framework [40], we posit that psychiatric emergency physicians prioritize the Principle of Beneficence over the Principle of Autonomy when formulating clinical decisions to ensure the safety of both patients and others. Most patients come to the hospital accompanied by their family members, who hope that the patients can receive timely treatment and care in the hospital. This might be another ethical considerations that doctors may take into account when making decisions. We should pay attention to the ethical requirements when implementing compulsory measures in PERs to reduce unnecessary medical disputes [42]. Setting up an internal review mechanism is a also feasible way [43]. Environmental modifications such as the"safe ward” intervention model can reduce the use of restraint measures [44].

Psychiatric emergency visits and low triage levels have increased, while hospital admissions and emergency observations stays have declined. The inadequate implementation of tiered diagnosis and treatment has resulted in a strain on psychiatric emergency medical resources. Owing to the availability of more treatment options and psychiatrists in tertiary specialized hospitals and the absence of a referral requirement, patients can seek medical treatment without being referred [45]. A WHO report emphasized that effective mental health reform requires a shift in focus from psychiatric hospitals to community-based services [2]. Evidence suggests that community treatment orders (CTOs) effectively enhance service continuity, reduce emergency visits, and mitigate violence [46]. However, the lack of specialized psychiatric wards in primary healthcare institutions and their limited capacity for initial diagnosis and treatment of mental disorders, tiered diagnosis and treatment cannot be effectively implemented. It is imperative to establish dedicated psychiatric inpatient units within secondary hospitals and invest in diagnostic training and infrastructure for primary psychiatric practitioners.

### The two-stage impact of public health crises on PES

The impact of public health crises on PES shows a two-stage trend, featuring both short-term fluctuations and long-term lag effects. During the pandemic, about 67% of countries experienced disruptions in psychological counseling and psychotherapy services and 30% of countries reported disruptions in access to medications for mental, neurological and substance use disorders [47]. Due to various factors such as social isolation and the reduced accessibility of mental health facilities, a review shows that the suicide behavior rate among patients with mental disorders has increased [48]. The impact brought by public health events is immediate, leading to a large number of psychiatric patients flocking to emergency departments for treatment.

The affiliated brain hospital, Guangzhou Medical University has also encountered the problems mentioned above. In 2020, the number of emergency visits and the post-hospital restrained rate increased significantly. A study in Shanghai, China, has also shown compared with the period before the epidemic, the number of visits to PED has been increasing [49]. However, the studies conducted in Boston [50] and Northern Israel [51] presented opposite results: the utilization rate of PES was in a declining state during the lockdown. A study in Berlin, Germany, shows that compared with the pre-COVID period, there was no significant change in the overall number of visits to PED and the overall number of hospitalizations during the COVID-19 pandemic [52]. We believe this might be related to the differences in medical service facilities, medical insurance policy and the public’s awareness of seeking medical treatment. Beside, the hospital admissions rate has decreased significantly in 2021. A study in Denmark [53] shows a similar trend of change. It highlighted the vulnerability of PES during public health events.

Barlattani T et al. [54] found that the potential impacts on mental health became evident following the complete lifting of restrictions and the restoration of normal local health management practices. Some of our research findings confirm this statement. This long-term effect is reflected in the delayed outbreak of diagnostic classifications. In our study, after lifting epidemic control measures (2023–2024), the proportion of emergency patients with F30-F39 and F40-F48 disorders increased continuously. Additionally, the teenager and female patients visits have continuously increased. A Chilean report shows women face increased household and childcare burdens and are more likely to lose jobs or income due to the pandemic [55].

The “lag effect” of the pandemic has a significant impact on the resource allocation of PES. We found that the voluntary and lower-level triage visits have continuously risen. During public health crises, primary psychological services (such as community clinics) are weakened due to personnel reallocation or suspension of offline operations, forcing lower-level demands to concentrate in emergency departments. The social and economic aftereffects of the epidemic, such as unemployment and the estrangement of interpersonal relationships, have given rise to a “regularly stressed psychological group”. Due to the behavioral inertia of the immediate availability of PES, emergency resources have been occupied by non-urgent demands. To fortify the resilience of PES in the face of public health events, our government ought to meticulously craft and implement tailored emergency response strategies.

### Limitations and suggestions for future research

Several limitations must be acknowledged in the present study. First of all, as this article is a retrospective study and the data source is from a single center, our results may be difficult to extrapolate to other populations or regions. Additionally, when collecting data, some psychiatric patients were reluctant to disclose their demographic variables (e.g.marital status), or due to factors such as the influence of diseases, it is impossible to obtain their demographic variables. We marked this part of the data as Unknown. Besides, this study only conducted basic comparative statistical analysis. In future, we plan to build a prediction model related to PES against the backdrop of public health crises, so as to be able to plan in advance and optimize resource allocation after the occurrence of public health crises, and enhance the recovery capacity and response efficiency of PES.

## Conclusion

PES in southern China have shown notable development between 2018 and 2024, as reflected by more voluntary health-seeking behaviors, broader coverage of medical insurance, more cautious restraint measures used, and more precise diagnoses. Despite these advancements, several challenges remain: PES requirements for under-served groups(unmarried, unemployed, higher education level and male) may be overlooked. Psychiatric referral services remain inadequate, leading to overburdened tertiary facilities. Disparities between judicial and psychiatric staff in applying coercive measures may raise ethical concerns. Public health events can have both immediate and long-term dual impacts on psychiatric emergency services. To address these issues, comprehensive reforms are necessary, including strengthening referral systems, enhancing community-based care, and improving ethical practices in coercive interventions. Reasonable policies should be formulated to better respond to public health crises. These measures will ensure that PES can adapt to the evolving needs of psychiatric patients and continue to improve service quality.

## Data Availability

No datasets were generated or analysed during the current study.
